# Probability Distributions Describing Qubit-State Superpositions

**DOI:** 10.3390/e25101366

**Published:** 2023-09-22

**Authors:** Margarita A. Man’ko, Vladimir I. Man’ko

**Affiliations:** 1 Lebedev Physical Institute, Leninskii Prospect 53, Moscow 119991, Russia; mankovi@lebedev.ru; 2 Department of Theoretical Physics, Moscow Institute of Physics and Technology, Dolgoprudnyi 141700, Russia; 3 Russian Quantum Center, Skolkovo, Moscow 143025, Russia

**Keywords:** probability distributions, tomographic probability distribution, Wigner function, Husimi function, entangled probability distribution, separable probability distribution, entropy

## Abstract

We discuss qubit-state superpositions in the probability representation of quantum mechanics. We study probability distributions describing separable qubit states. We consider entangled states on the example of a system of two qubits (Bell states) using the corresponding superpositions of the wave functions associated with these states. We establish the connection with the properties and structure of entangled probability distributions.

## 1. Introduction

The conventional probability theory plays a crucial role in considering quantum phenomena [1,2,3,4]. In connection with the development of quantum mechanics and discovery of such phenomena as entanglement and Bell inequality [5,6], the role of conditional probabilities was discussed in [7], where it was associated with quantum formalism for dichotomic probabilities. The other aspects of Bell states were discussed in [8].

In conventual formulation of quantum mechanics, the quantum system states are associated with the vectors ∣ψ〉 in a Hilbert space H [9] (for pure states) or with the density operators (for mixed or pure states) $\hat{\rho }$ [10,11] acting in the Hilbert space. The vectors ∣ψ〉 or operators $\hat{\rho }$ are described by the wave functions $\psi \left(x\right)$ or density matrices $\rho \left(x,x^{\prime }\right)$ in the position representation or other representations, like the Wigner function [12]. Stratonovich introduced an analogous representation of spin state using a concept of the operator [13], later on known as quantizer [14]. The wave functions or density matrices provide the possibility to obtain probability distributions, either $|\psi \left(x\right){|}^{2}$ or $\rho \left(x,x\right)$. Recently, the probability representation of quantum mechanics was constructed [15] for systems with continuous variables like oscillator or systems with discrete variables like spin-1/2 or qubits [16]. The construction of probability representations was developed using the concept of quantizer and dequantizer [17,18]. Some properties of the probability distributions used in quantum mechanics were discussed in [19,20,21,22,23].

The probability representation of quantum states is based on the possibility to construct an invertible map of density operators $\hat{\rho }$ acting in a Hilbert space H onto the probability distribution functions describing all physical properties of quantum states, which, in turn, are described by the conventional formalism of wave functions or density matrices both for systems with continuous variables and systems with discrete variables. This means that, for a known operator $\hat{\rho }$ of any quantum system state, there exists the probability distribution function *w*, i.e., $\hat{\rho }⟷w$ containing the same information on the system state, which is available in the density operator $\hat{\rho }$. For oscillator’s system states, including the entangled states, the probability distributions were studied in [24,25].

Thus, quantum mechanics provides the possibility to study new properties of probability distributions that were not discussed in the literature. There was no use of the probability theory associated with quantum formalism because the classical science did not consider such notions as the wave function or the Hilbert space vectors and density operators. The aim of our work is to consider some aspects of probability distributions describing some properties of qubit states and their relations to the superposition principle of quantum mechanics. We consider a simple example of the entangled state of two qubits (Bell state) in the probability representation of quantum states. We show that the superpositions of two-qubit state vectors describing the spin projections in the opposite directions of *x*, *y*, and *z* axes can be described by the conditional probability distributions of two random dichotomic variables. This probability distribution, called the entangled probability distribution, is related to the probability distribution describing the two-qubit state associated with a separable state, for which the density matrix is a convex sum of two terms of tensor products of two density matrices of each spin-1/2 state. The structure of entangled probability distribution reflects the difference in the spin-state superposition and an extra term containing the influence of correlations available in entangled quantum states. We construct explicit forms of the conditional probability distributions of entangled and separable states and express the purity parameter of these states in terms of the probabilities describing these states. Also, we establish the rule of a group-like product of probability distributions in view of the multiplication rule of density operators.

This paper is organized as follows.

In Section 2, we discuss the formalism of quantizer–dequantizer operators acting in the Hilbert space of qubit states to be used for constructing the probability distributions describing both entangled and separable two-qubit states. In Section 3, the qubit probability distributions are constructed. Explicit expressions for dequantizer operators for two-qubit states are obtained in Section 4. The unitary transforms of these probability distributions are studied in Section 5. Then, in Section 6, we consider the superposition state vectors in the Hilbert space and the structure of probability distributions describing the entangled states. Section 7 and Section 8 are devoted to entangled two-qubit states in the probability representation. The relations of introduced separable probability distribution and entangled probability distribution are discussed in Section 9. Finally, the conclusions and prospectives are provided in Section 10.

## 2. Hilbert Spaces and Quantizer–Dequantizer Operator Formalism

To describe the probability representation of qubit and qudit states, first we present the method of an invertible map of operators $\hat{A}$, acting on vectors in a Hilbert space H, onto functions $f_{\hat{A}}\left(\vec{X}\right)$ using two families of operators $\hat{U}\left(\vec{X}\right)$ and $\hat{D}\left(\vec{X}\right)$, acting in the same Hilbert space H, where $\vec{X}=\left(X_{1},X_{2},\ldots ,X_{N}\right)$ and parameters ${\vec{X}}_{k}$; k=1,2,…,N can be either discrete or continuous ones [17,18].

Assume that we found the following relations:

(1) $$\begin{array}{c} f_{\hat{A}}\left(\vec{X}\right)=\mathrm{Tr}\;\hat{A}\hat{U}\left(\vec{X}\right), \end{array}$$

(2) $$\begin{array}{c} \hat{A}=\int f_{\hat{A}}\left(\vec{X}\right)\hat{D}\left(\vec{X}\right)\;d\vec{X}. \end{array}$$

Operators $\hat{U}\left(\vec{X}\right)$ map operators onto functions and they are called dequantizers, while operators $\hat{D}\left(\vec{X}\right)$ map functions onto operators and they are called quantizers.

In the case where functions $f_{\hat{A}}\left(\vec{X}\right)$ for operators $\hat{A}$ turn out to be probability distribution functions, we call the representation of the operators $\hat{A}$ the probability representation.

If the operators $\hat{A}$ have the properties of density operators, i.e., ${\hat{\rho }}^{†}=\hat{\rho }$, $\mathrm{Tr}\;\hat{\rho }=1$, and all the eigenvalues of the operator $\hat{A}$ are nonnegative, and the dequantizer operators $\hat{U}\left(\vec{X}\right)$ have the same properties, we arrive at the probability representation of operators $\hat{A}$, in view of Born’s rule [26,27,28]. The vector $\vec{X}$ can contain parameters of random variables and condition parameters for the case of the probability representation of the density operator $\hat{\rho }$.

In the cases where discussed above properties of operators $\hat{U}\left(\vec{X}\right)$ are violated, we have other representations of density operators $\hat{\rho }=\hat{A}$, like the Wigner representation [12], or the quasiprobability representations, like the Husimi quasiprobability representation [29] and Glauber–Sudarshan quasiprobability representation [30,31].

In this paper, we consider the two-dimensional Hilbert spaces (examples of qubits) and four-dimensional Hilbert spaces (examples of ququarts); for infinite-dimensional probability representations (tomograms), see [32].

Assume that we have three operators $\hat{U}\left(\vec{X}\right)$, namely

(3) $$\begin{array}{c} \hat{U}\left(1\right)=\left(\begin{array}{cc} 1 & 0\\ 0 & 0 \end{array}\right),\;\hat{U}\left(2\right)=\left(\begin{array}{cc} 1/2 & 1/2\\ 1/2 & 1/2 \end{array}\right),\;\hat{U}\left(3\right)=\left(\begin{array}{cc} 1/2 & -i/2\\ i/2 & 1/2 \end{array}\right), \end{array}$$

which have the properties of density operators and, due to this reason, an arbitrary density 2 × 2 matrix

(4) $$\begin{array}{c} \hat{\rho }=\left(\begin{array}{cc} \rho_{11} & \rho_{12}\\ \rho_{21} & \rho_{22} \end{array}\right) \end{array}$$

can be expressed as follows:

(5) $$\begin{array}{c} \hat{\rho }=\left(\begin{array}{cc} p_{3} & \left(p_{1}-1/2\right)-i\left(p_{2}-1/2\right)\\ \left(p_{1}-1/2\right)+i\left(p_{2}-1/2\right) & 1-p_{3} \end{array}\right), \end{array}$$

where the numbers $p_{1}$, $p_{2}$, and $p_{3}$ are nonnegative and correspond to the probability distributions of dichotomic random spin-1/2 projections.

## 3. Qubit Probability Distributions

We introduce conditional probability distributions describing qubit states using the notation $w\left(X|j\right)$, where parameter *X* can take two values X=+1/2,−1/2 and parameter *j* takes three values j=1,2,3. Parameter *X* describes the spin-1/2 projection ±1/2 onto three perpendicular directions *x*, *y*, and *z*. Thus, the spin-1/2 state is described by the conditional probability distribution $w\left(X|j\right)$, where nonnegative numbers $0\leq p_{1},p_{2},p_{3}\leq 1$ are given by the functions

(6) $$\begin{array}{c} w\left(+1/2|1\right)=p_{1},\;w\left(+1/2|2\right)=p_{2},\;w\left(+1/2|3\right)=p_{3},\\ w\left(-1/2|1\right)=1-p_{1},\;w\left(-1/2|2\right)=1-p_{2},\;w\left(-1/2|3\right)=1-p_{3}. \end{array}$$

These numbers satisfy the condition

(7) $${\left(p_{1}-1/2\right)}^{2}+{\left(p_{2}-1/2\right)}^{2}+{\left(p_{3}-1/2\right)}^{2}\leq 1/4,$$

corresponding to the properties of Hermitian density matrix ρ of the qubit state, which has nonnegative eigenvalues and Tr ρ=1.

One can see that there exists the relation between the quantizer–dequantizer approach and the generalized Bloch decomposition of an operator on a finite-dimensional Hilbert space through an operator basis; see [33]. In particular, there is a direct connection between the qubit parameters $p_{j}$; j=1,2,3 and the components of the Bloch vector of a qubit state in its Bloch representation. Also, analogous relations can be found for probabilities describing ququart states with the qudit state approach developed in [34,35].

Thus, we associate the qubit state with the conditional probability distribution $w\left(X|j\right)$, where parameter *j* corresponds to the direction of the axes x,y, and *z* in the space. Different kinds of the conditional probability distributions were found in [16], where number of conditions were infinite.

Also, we can introduce the joint probability distribution $W\left(X,j\right)=w\left(X|j\right)\Pi \left(j\right)$, where the nonnegative function Π(j) has the properties $\sum_{j=1}^{3}\Pi \left(j\right)=1$; i.e., it is the probability distribution of random directions of three axes x,y, and *z* in the space. So, we have

(8) $$\sum_{j=1}^{3}W\left(+1/2,j\right)+W\left(-1/2,j\right)=1.$$

We can choose the distribution Π(j)=1/3. For all possible distributions Π(j), the density matrix ρ has the same form.

Thus, we introduced probability distributions $w\left(X|j\right)$ (6) and $W\left(X,j\right)$, which contain the same information on qubit state as available in the density matrix $\hat{\rho }$ (4).

### 3.1. Conditional Probability Distributions of the Pure Qubit State

Now, we consider the pure qubit state vector of the form

(9) $$|\psi \rangle =\left(\begin{array}{c} \psi_{1}\\ \psi_{2} \end{array}\right)\cdot \frac{1}{\sqrt{|\psi_{1}{|}^{2}+{\left|\psi_{2}\right|}^{2}}}\;.$$

For the density matrix $\rho_{\psi }$ of the pure state ∣ψ〉, namely $\hat{\rho }=|\psi \rangle \langle \psi |$, we have

(10) $$\hat{\rho }=\left(\begin{array}{cc} \psi_{1}\psi_{1}^{*} & \psi_{1}\psi_{2}^{*}\\ \psi_{2}\psi_{1}^{*} & \psi_{2}\psi_{2}^{*} \end{array}\right)\cdot \frac{1}{|\psi_{1}{|}^{2}+{\left|\psi_{2}\right|}^{2}}\;;$$

thus, we obtain $p_{3}=\frac{\psi_{1}\psi_{1}^{*}}{|\psi_{1}{|}^{2}+{\left|\psi_{2}\right|}^{2}}$. The density operator has the properties

(11) $${\hat{\rho }}^{†}=\hat{\rho },\;\mathrm{Tr}\;\hat{\rho }=1,\;{\hat{\rho }}^{2}=\hat{\rho }.$$

Also, the purity parameter μ of state (9) reads $\mu =\mathrm{Tr}\;\rho_{\psi }^{2}=1$.

### 3.2. Product of Probability Distributions of Two-Qubit States

We calculate probability distributions of two products of qubit-state density operators; they read

(12) $$\left(\begin{array}{cc} p_{3} & p^{*}\\ p & 1-p_{3} \end{array}\right)\cdot \left(\begin{array}{cc} P_{3} & P^{*}\\ P & 1-P_{3} \end{array}\right)=\left(\begin{array}{cc} p_{3}P_{3}+p^{*}P & p_{3}P^{*}+p^{*}\left(1-P_{3}\right)\\ pP_{3}+\left(1-p_{3}\right)P & pP^{*}+\left(1-p_{3}\right)\left(1-P_{3}\right) \end{array}\right).$$

We introduce the notion of a group-like product of two probability distributions $w\left(X|j\right)$ and $w\left(Y|k\right)$, in view of (6) and (7); this rule is expressed in terms of the anticommutator

(13) $$\frac{1}{N}\left\{\left(\begin{array}{cc} p_{3} & p^{*}\\ p & 1-p_{3} \end{array}\right),\left(\begin{array}{cc} P_{3} & P^{*}\\ P & 1-P_{3} \end{array}\right)\right\}=\left(\begin{array}{cc} \Pi_{3} & \Pi^{*}\\ \Pi & 1-\Pi_{3} \end{array}\right).$$

Here, *N* is the trace of anticommutator, and numbers $\Pi =\left(\Pi_{1}-1/2\right)+i\left(\Pi_{2}-1/2\right)$ are the probabilities determining the qubit density matrix $\rho_{\Pi }=\frac{1}{N}\left({\hat{\rho }}_{p}{\hat{\rho }}_{P}+{\hat{\rho }}_{P}{\hat{\rho }}_{p}\right)$ in the probability representation of qubit states. The introduced product of probability distributions, determining the product of density matrices, is similar to the product of group elements; thus, we arrive at

(14) $$\Pi_{3}=2\left[p_{3}P_{3}+\frac{1}{2}\left(p^{*}P+P^{*}p\right)\right]\frac{1}{N},\;\Pi =\left(p+P\right)\frac{1}{N}.$$

Formula (6) provides the rule of how to express a group-like multiplication of probability distributions $w_{1}\left(X|j\right)$, $w_{2}\left(X|j\right)$, and $w_{3}\left(X|j\right)$ corresponding to products of probability values $p_{1},p_{2},p_{3}$ and $P_{1},P_{2},P_{3}$ and providing the probabilities $\Pi_{1},\Pi_{2},\Pi_{3}$. In the state $\rho_{\Pi }$, the density matrices must be Hermitian and have the trace equal to one; these constraints are satisfied by the construction of matrix $\rho_{\Pi }$, but they must have only nonnegative eigenvalues. The example of such possibility is matrix $\rho^{2}=\frac{1}{N}\left(\rho \rho +\rho \rho \right)$, where $\rho_{\Pi }=\frac{1}{N}\rho^{2}$ for matrix ρ, which is the density matrix of qubit state with the probability distribution determined by the probability numbers $p_{1},p_{2},p_{3}$ and $P_{1}=p_{1},P_{2}=p_{2},P_{3}=p_{3}$.

For pure states, $\rho^{2}=\rho$.

For mixed states, the normalization constant $N=\mathrm{Tr}\;\rho^{2}=\mu$ is called the purity parameter. The purity parameter in the probability representation of quantum qubit state reads

(15) $$\mu =2\left(p_{1}^{2}+p_{2}^{2}+p_{3}^{2}-p_{1}-p_{2}-p_{3}+1\right);$$

it is a new characteristic of the state expressed in terms of probabilities describing the states.

The other explicit example of the dependence of probabilities $\Pi_{1},\Pi_{2},\Pi_{3}$ on probabilities $p_{1},p_{2},p_{3}$ and $P_{1},P_{2},P_{3}$ can be obtained for density matrices $\rho_{p}$ and $\rho_{P}$ of qubit states, which may simultaneously be diagonalyzed and commute. This means that we have an explicit formula for the dependence of probabilities $\Pi_{1},\Pi_{2},\Pi_{3}$ as the functions of probabilities $p_{1},p_{2},p_{3}$ and $P_{1},P_{2},P_{3}$ and the trace of matrix $\rho_{\Pi }^{2}={\left[\frac{1}{N}\left(\rho_{p}\rho_{P}+\rho_{P}\rho_{p}\right)\right]}^{2}$. The previous example is the partial case of this one where $\rho_{p}=\rho_{P}$, i.e., $p_{j}=P_{j}$. Other cases take place for the situations where matrix $\rho_{p}\rho_{P}+\rho_{P}\rho_{p}$ has nonnegative eigenvalues.

Also, we introduce the density matrix of the direct product of density matrices of two-qubit states in the probability representation,

(16) $$\left(\begin{array}{cc} p_{3} & p^{*}\\ p & 1-p_{3} \end{array}\right)⊗\left(\begin{array}{cc} P_{3} & P^{*}\\ P & 1-P_{3} \end{array}\right)=\left(\begin{array}{cc} p_{3}\left(\begin{array}{cc} P_{3} & P^{*}\\ P & 1-P_{3} \end{array}\right) & p^{*}\left(\begin{array}{cc} P_{3} & P^{*}\\ P & 1-P_{3} \end{array}\right)\\ p\left(\begin{array}{cc} P_{3} & P^{*}\\ P & 1-P_{3} \end{array}\right) & \left(1-p_{3}\right)\left(\begin{array}{cc} P_{3} & P^{*}\\ P & 1-P_{3} \end{array}\right) \end{array}\right).$$

Thus, for our example, we obtain the equations for matrix elements of ququart density matrix,

(17) $$\begin{array}{c} 1=p_{3}P_{3},\;p_{3}P^{*}=0,\;p^{*}P_{3}=0,\;p^{*}P^{*}=0,\\ 0=p_{3}P,\;p_{3}\left(1-P\right)=0,\;p^{*}P=0,\;p^{*}\left(1-P_{3}\right)=0; \end{array}$$

in both matrices, there is only one nonzero matrix element, with $p_{3}=P_{3}=1$.

### 3.3. Notation for Separable and Entangled Probability Distributions

Now, we discuss the notation for probability distributions of dichotomic random variables. For a single qubit state, we use the notation of conditional probability distribution, namely random parameter *X* takes two values +1/2 and −1/2 and the condition parameters take three values j,k=1,2,3.

For two qubits, we adopt the conditional probability distribution function $w\left(X,Y|j,k\right)$, where random parameters X=±1/2, Y=±1/2, and condition parameters j,k=1,2,3. The real nonnegative conditional probability distributions satisfy the normalization condition

(18) $$\sum_{X}\sum_{Y}w\left(X,Y|j,k\right)=1.$$

For quqauart state, there are probability distributions determined by probabilities $p_{1},p_{2},p_{3}$ and $P_{1},P_{2},P_{3}$, which provide the density matrices of the form giving separable and entangled probability distributions determining the states.

## 4. Dequantizer Operator $\hat{U}\left(X|j\right)$ for Qubit State

Let us check that one can obtain the dequantizer operator for the qubit state, with the density 2 × 2-matrix $\hat{\rho }$ determining the probability distribution $w\left(X|j\right)$ for dichotomic random variables, which take values X=±1/2, i.e., spin-1/2 projections on three axes *x*, *y*, and *z* in the space labeled by numbers j=1,2,3 called the condition parameters. For this, we consider normalized eigenvectors of Pauli matrices

(19) $$\sigma_{x}=\left(\begin{array}{cc} 0 & 1\\ 1 & 0 \end{array}\right),\;\sigma_{y}=\left(\begin{array}{cc} 0 & -i\\ i & 0 \end{array}\right),\;\sigma_{z}=\left(\begin{array}{cc} 1 & 0\\ 0 & -1 \end{array}\right).$$

Their normalized eigenvectors determining the spin-1/2 states are eigenstates of operators – spin-1/2 projections ${\hat{s}}_{x}=\frac{1}{2}\;\sigma_{x}$, ${\hat{s}}_{y}=\frac{1}{2}\;\sigma_{y}$, and ${\hat{s}}_{z}=\frac{1}{2}\;\sigma_{z}$, namely

(20) $$|\psi_{x+}\rangle =\left(\begin{array}{c} \frac{1}{\sqrt{2}}\\ \frac{1}{\sqrt{2}} \end{array}\right),\;|\psi_{y+}\rangle =\left(\begin{array}{c} \frac{1}{\sqrt{2}}\\ \frac{i}{\sqrt{2}} \end{array}\right),\;|\psi_{z+}\rangle =\left(\begin{array}{c} 1\\ 0 \end{array}\right),$$

such that

(21) $${\hat{s}}_{x}|\psi_{x+}\rangle =\frac{1}{2}|\psi_{x+}\rangle ,\;{\hat{s}}_{y}|\psi_{y+}\rangle =\frac{1}{2}|\psi_{y+}\rangle ,\;{\hat{s}}_{z}|\psi_{z+}\rangle =\frac{1}{2}|\psi_{z+}\rangle .$$

Also, we have the other eigenstates – vectors

(22) $$|\psi_{x-}\rangle =\left(\begin{array}{c} \frac{1}{\sqrt{2}}\\ -\frac{1}{\sqrt{2}} \end{array}\right),\;|\psi_{y-}\rangle =\left(\begin{array}{c} \frac{1}{\sqrt{2}}\\ -\frac{i}{\sqrt{2}} \end{array}\right),\;|\psi_{z-}\rangle =\left(\begin{array}{c} 0\\ 1 \end{array}\right),$$

for which the spin projections on three axes *x*, *y*, and *z* in the space are equal to −1/2.

Thus, we have three density operators $\hat{\rho }\left(+1/2|j\right)\equiv \hat{U}\left(+1/2|j\right)$; j=1,2,3. They read

(23) $$\hat{U}\left(+1/2|1\right)=\left(\begin{array}{cc} 1/2 & 1/2\\ 1/2 & 1/2 \end{array}\right),\;\hat{U}\left(+1/2|2\right)=\left(\begin{array}{cc} 1/2 & -i/2\\ i/2 & 1/2 \end{array}\right),\;\hat{U}\left(+1/2|3\right)=\left(\begin{array}{cc} 1 & 0\\ 0 & 0 \end{array}\right)$$

and satisfy the properties of spin-state density operators describing the pure states of the spin with spin projections on three axes *x*, *y*, and *z* in the space equal to +1/2.

Also, we have three density operators $\hat{\rho }\left(-1/2|j\right)\equiv \hat{U}\left(-1/2|j\right)$; j=1,2,3. They read

(24) $$\hat{U}\left(-1/2|1\right)=\left(\begin{array}{cc} 1/2 & -1/2\\ -1/2 & 1/2 \end{array}\right),\;\hat{U}\left(-1/2|2\right)=\left(\begin{array}{cc} 1/2 & i/2\\ -i/2 & 1/2 \end{array}\right),\;\hat{U}\left(-1/2|3\right)=\left(\begin{array}{cc} 0 & 0\\ 0 & 1 \end{array}\right)$$

and satisfy the properties of spin-state density operators describing the pure states of the spin with spin projections on three axes *x*, *y*, and *z* in the space equal to −1/2.

In view of Born’s rule, numbers $\mathrm{Tr}\left[\hat{\rho }\left(+1/2|j\right)\hat{\rho }\right]$ and $\mathrm{Tr}\left[\hat{\rho }\left(-1/2|j\right)\hat{\rho }\right]$ for an arbitrary density operator $\hat{\rho }$ are probabilities, i.e.,

(25) $$\begin{array}{c} w\left(+1/2|j\right)=\mathrm{Tr}\left[\hat{\rho }\;\hat{U}\left(+1/2|j\right)\right], \end{array}$$

(26) $$\begin{array}{c} w\left(-1/2|j\right)=\mathrm{Tr}\left[\hat{\rho }\;\hat{U}\left(-1/2|j\right)\right]. \end{array}$$

One can check that these conditional probabilities are equal to numbers $p_{1}$, $1-p_{1}$, $p_{2}$, $1-p_{2}$, and $p_{3}$, $1-p_{3}$ determining the spin states, with the density matrix $\hat{\rho }$ of the form

(27) $$\hat{\rho }=\left(\begin{array}{cc} p_{3} & \left(p_{1}-1/2\right)-i\left(p_{2}-1/2\right)\\ \left(p_{1}-1/2\right)+i\left(p_{2}-1/2\right) & 1-p_{3} \end{array}\right).$$

For pure states, ${\hat{\rho }}^{2}=\hat{\rho }$, the probabilities $p_{1}$, $p_{2}$, and $p_{3}$, as well as $w\left(\pm 1/2|j\right)$, satisfy the condition given by the following matrix relation:

(28) $$\left(\begin{array}{cc} p_{3} & \Delta^{*}\\ \Delta & 1-p_{3} \end{array}\right)\cdot \left(\begin{array}{cc} p_{3} & \Delta^{*}\\ \Delta & 1-p_{3} \end{array}\right)=\left(\begin{array}{cc} p_{3} & \Delta^{*}\\ \Delta & 1-p_{3} \end{array}\right),$$

where $\Delta =\frac{1}{2}\left[w\left(+1/2|1\right)+i\;w\left(+1/2|2\right)\right]-\frac{1}{2}\left[w\left(-1/2|1\right)+i\;w\left(-1/2|2\right)\right]$,

$p_{3}=w\left(+1/2|3\right)$, and $\;1-p_{3}=w\left(-1/2|3\right)$.

## 5. Unitary Transforms of Probability Distributions Determining Spin States

The above presented description of spin states by conditional probability distributions $w\left(X|j\right)$ demonstrates that the probability distributions can be used to introduce their unitary transforms corresponding to unitary transforms of the spin-state density operators.

The Schrödinger and von Neumann equations for density operators $\hat{\rho }\left(t\right)$ of spin states

(29) $$\frac{\partial \hat{\rho }\left(t\right)}{\partial t}+i\left[\hat{H},\rho \left(t\right)\right]=0$$

have the solution of the form

(30) $$\hat{\rho }\left(t\right)=e^{-i\hat{H}t}\hat{\rho }\left(0\right)\;e^{i\hat{H}t},$$

where $\hat{H}$ is Hermitian Hamiltonian, and 2 × 2-matrix $\hat{u}=e^{-i\hat{H}t}$ provides the possibility to introduce the unitary transform of conditional probability distributions given by the following matrix relationship:

(31) $$\begin{array}{c} \left(\begin{array}{cc} u_{11} & u_{12}\\ u_{21} & u_{22} \end{array}\right)\left(\begin{array}{cc} w\left(+1/2|3\right) & \Delta^{*}\\ \Delta & w\left(-1/2|3\right) \end{array}\right)\left(\begin{array}{cc} u_{11}^{*} & u_{21}^{*}\\ u_{12}^{*} & u_{22}^{*} \end{array}\right)\\ =\left(\begin{array}{cc} w\left(+1/2|3,t\right) & \Delta^{*}\left(t\right)\\ \Delta (t) & w\left(-1/2|3,t\right) \end{array}\right), \end{array}$$

where probabilities $w\left(\pm 1/2|j,t\right)$ are obtained as transformed conditional probabilities $w\left(\pm 1/2|j\right)$ and

$$\Delta \left(t\right)=\frac{1}{2}\left[w\left(+1/2|1,t\right)-i\;w\left(-1/2|1,t\right)\right]+\frac{1}{2}\left[w\left(+1/2|2,t\right)-i\;w\left(-1/2|2,t\right)\right].$$

Thus, we introduced the notion of unitary transforms of conditional probability distributions $w\left(\pm 1/2|j\right)$, which follows from the formalism of quantum mechanics of the spin system evolution and has not been considered in the literature.

For example, we have the probability $w\left(+1/2|3,t\right)$, which reads

(32) $$u_{11}^{*}\left[u_{11}w\left(+1/2|3\right)+u_{12}\Delta \right]+u_{12}^{*}\left[u_{11}\Delta^{*}+u_{12}w\left(-1/2|3\right)\right]=w\left(+1/2|3,t\right).$$

## 6. Superposition of Probabilities

Let us obtain the probability distribution of the pure qubit state given by the superposition principle. Given two spin states with normalized state vectors $|\psi_{1}\rangle$ and $|\psi_{2}\rangle$, i.e.,

$C_{1}|\psi_{1}\rangle \;+\;C_{2}|\psi_{2}\rangle \;=\;|\psi \rangle$ and $\langle \psi_{1}|\psi_{1}\rangle =\langle \psi_{2}|\psi_{2}\rangle =\langle \psi |\psi \rangle =1$, the probability distribution $w\left(X|j\right)$ of state $\hat{\rho }=|\psi \rangle \langle \psi |$ is then given by the relation

(33) $$w\left(X|j\right)=\mathrm{Tr}\left[\hat{U}\left(X|j\right)\hat{\rho }\right]=\langle \psi |\hat{U}\left(X|j\right)|\psi \rangle .$$

Also, the probability distribution is expressed in terms of normalized vectors $|\psi_{1}\rangle =\left(\begin{array}{c} a\\ b \end{array}\right)$ and $|\psi_{2}\rangle =\left(\begin{array}{c} A\\ B \end{array}\right)$ as well as in terms of complex numbers $C_{1}$ and $C_{2}$ as follows:

(34) $$\begin{array}{ccc} w\left(X|j\right) & = & |C_{1}{|}^{2}\langle \psi_{1}|\hat{U}\left(X|j\right)|\psi_{1}\rangle +{\left|C_{2}\right|}^{2}\langle \psi_{2}|\hat{U}\left(X|j\right)|\psi_{2}\rangle \\ & & +\;\;C_{1}^{*}C_{2}\langle \psi_{1}|\hat{U}\left(X|j\right)|\psi_{2}\rangle +C_{2}^{*}C_{1}\langle \psi_{2}|\hat{U}\left(X|j\right)|\psi_{1}\rangle . \end{array}$$

For $|\psi_{1}\rangle =\left(\begin{array}{c} a\\ b \end{array}\right)$ and $|\psi_{2}\rangle =\left(\begin{array}{c} A\\ B \end{array}\right)$, in terms of parameters *a*, *b* and *A*, *B*, with properties ${\left|a\right|}^{2}+{\left|b\right|}^{2}={\left|A\right|}^{2}+{\left|B\right|}^{2}=1$, we arrive at the superposition rule for probabilities $w\left(X|j\right)$, where X=+1/2,−1/2; j=1,2,3, determined by complex parameter $C_{1}$ and $C_{2}$ such that 〈ψ∣ψ〉=1. It reads

(35) $$\begin{array}{ccc} w\left(X|j\right) & = & |C_{1}{|}^{2}\left(a^{*},b^{*}\right)\hat{U}\left(X|j\right)\left(\begin{array}{c} a\\ b \end{array}\right)+{\left|C_{2}\right|}^{2}\left(A^{*},B^{*}\right)\hat{U}\left(X|j\right)\left(\begin{array}{c} A\\ B \end{array}\right)\\ & & +\;C_{1}^{*}C_{2}\left(a^{*},b^{*}\right)\hat{U}\left(X|j\right)\left(\begin{array}{c} A\\ B \end{array}\right)+C_{2}^{*}C_{1}\left(A^{*},B^{*}\right)\hat{U}\left(X|j\right)\left(\begin{array}{c} a\\ b \end{array}\right). \end{array}$$

Here, we use the standard multiplication rule of rectangular and quadratic matrices to obtain the probabilities $w\left(X|j\right)$ determining the superposition state $C_{1}|\psi_{1}\rangle \;+\;C_{2}|\psi_{2}\rangle$, which depends on parameters *a*, *b* and *A*, *B*, as well as $C_{1}$ and $C_{2}$.

For a single state $|\psi_{1}\rangle =\left(\begin{array}{c} a\\ b \end{array}\right)$, the probability distribution $w_{1}\left(X|j\right)$ reads

(36) $$w_{1}\left(X|j\right)=\left(a^{*},b^{*}\right)\hat{U}\left(X|j\right)\left(\begin{array}{c} a\\ b \end{array}\right).$$

Also, for a single state $|\psi_{2}\rangle =\left(\begin{array}{c} A\\ B \end{array}\right)$, the probability distribution $w_{2}\left(X|j\right)$ is

(37) $$w_{2}\left(X|j\right)=\left(A^{*},B^{*}\right)\hat{U}\left(X|j\right)\left(\begin{array}{c} A\\ B \end{array}\right).$$

For the superposition of these two states, we have additional terms; see (35).

For the discussed pure states, the obtained probability distributions $w\left(X|j\right)$ satisfy the condition ${\hat{\rho }}_{\psi }^{2}={\hat{\rho }}_{\psi }$, where the pure-state operators ${\hat{\rho }}_{\psi }$ are determined by the probability distributions.

## 7. Probability Distributions Determined by Different Pairs of Quantizer–Dequantizer Operators

The formalism of different pairs of quantizer–dequantizer operators, ${\hat{U}}_{1}\left(\vec{X}\right)$, ${\hat{D}}_{1}\left(\vec{X}\right)$ and ${\hat{U}}_{2}\left(\vec{Y}\right)$, ${\hat{D}}_{2}\left(\vec{Y}\right)$, such that the state density operator ${\hat{\rho }}_{w_{12}}$, with matrix elements ${\hat{\rho }}_{\alpha \beta }^{\left(1\right)}$ and ${\hat{\rho }}_{\gamma \delta }^{\left(2\right)}$, reading either as

(38) $${\hat{\rho }}_{w_{1}}^{\left(1\right)}=\int w_{1}\left(\vec{X}\right){\hat{D}}_{1}\left(\vec{X}\right)\;d\vec{X}$$

or as

(39) $${\hat{\rho }}_{w_{2}}^{\left(1\right)}=\int w_{2}\left(\vec{Y}\right){\hat{D}}_{2}\left(\vec{Y}\right)\;d\vec{Y},$$

provides the possibility to introduce the relation of functions $w_{1}\left(\vec{X}\right)$ and $w_{2}\left(\vec{Y}\right)$, which are symbols of the density operator $\hat{\rho }$ corresponding to dequantizers ${\hat{U}}_{1}\left(\vec{X}\right)$ and ${\hat{U}}_{2}\left(\vec{Y}\right)$.

In the case of dequantizers, which have the properties of density matrices, the possibility arises to introduce the probability distributions, which are symbols $w_{1}\left(\vec{X}\right)$ and $w_{2}\left(\vec{Y}\right)$.

If dequantizer ${\hat{U}}_{1}\left(\vec{X}\right)$ does not have the properties of density operators, symbols $w_{1}\left(\vec{X}\right)$ are not the probability distributions. In this case, there exists the relation that provides the possibility to express the probability distribution $w_{2}\left(\vec{Y}\right)$ in terms of symbols of the density operator $w_{1}\left(\vec{X}\right)$, namely

(40) $$w_{2}\left(\vec{Y}\right)=\int w_{1}\left(\vec{X}\right)\mathrm{Tr}\left[{\hat{D}}_{1}\left(\vec{X}\right)U_{2}\left(\vec{Y}\right)\right]\;d\vec{X}.$$

This is a new method to construct probability distribution functions.

Now, we consider the example of pure qubit states ∣ψ〉 such that 〈ψ∣ψ〉=1, where $|\psi \rangle =\left(\begin{array}{c} a\\ b \end{array}\right)$ and ${\left|a\right|}^{2}+{\left|b\right|}^{2}=1$, with ${\hat{\rho }}^{2}=|\psi \rangle \langle \psi |$. Then, the introduced probability distributions $w_{\psi }\left(X|j\right)$, with $X=\left(+1/2,-1/2\right)$; j=1,2,3, read

(41) $$\begin{array}{ccc} w_{\psi }\left(+1/2|1\right) & = & \left(a^{*},b^{*}\right)\left(\begin{array}{cc} 1/2 & 1/2\\ 1/2 & 1/2 \end{array}\right)\left(\begin{array}{c} a\\ b \end{array}\right)=\frac{1}{2}+\frac{a^{*}b+ab^{*}}{2},\\ w_{\psi }\left(+1/2|2\right) & = & \left(a^{*},b^{*}\right)\left(\begin{array}{cc} 1/2 & -i/2\\ i/2 & 1/2 \end{array}\right)\left(\begin{array}{c} a\\ b \end{array}\right)=\frac{1}{2}-\frac{i(a^{*}b-ab^{*})}{2},\\ w_{\psi }\left(+1/2|3\right) & = & \left(a^{*},b^{*}\right)\left(\begin{array}{cc} 1 & 0\\ 0 & 0 \end{array}\right)\left(\begin{array}{c} a\\ b \end{array}\right)=a^{*}a,\\ w_{\psi }\left(-1/2|1\right) & = & \frac{1}{2}-\frac{a^{*}b+b^{*}a}{2},\\ w_{\psi }\left(-1/2|2\right) & = & \frac{1}{2}+\frac{i(a^{*}b-b^{*}a)}{2},\\ w_{\psi }\left(-1/2|3\right) & = & b^{*}b. \end{array}$$

The obtained probabilities determine the probability distributions describing the qubit states in terms of their wave functions. Analogous relations for continuous variables, in the case of oscillator state, were found in [36], where the tomographic probability distribution of the state was expressed in terms of the Radon transform of its wave function.

The numbers *a* and *b* satisfy the condition for pure-state density operators ${\hat{\rho }}_{\psi }^{2}={\hat{\rho }}_{\psi }$, i.e.,

(42) $$\left(\begin{array}{cc} a^{*}a & ab^{*}\\ a^{*}b & b^{*}b \end{array}\right)\left(\begin{array}{cc} a^{*}a & ab^{*}\\ a^{*}b & bb^{*} \end{array}\right)=\left(\begin{array}{cc} a^{*}a & ab^{*}\\ a^{*}b & bb^{*} \end{array}\right);$$

this property means the corresponding equality for the conditional probability distribution $w_{\psi }\left(X|j\right)$ since

(43) $$\begin{array}{ccc} a^{*}b+b^{*}a=1-2\;w_{\psi }\left(-1/2|1\right), & & i\left(a^{*}b-ab^{*}\right)=2\;w_{\psi }\left(-1/2|2\right)-1,\\ a^{*}a=w_{\psi }\left(+1/2|3\right), & & b^{*}b=w_{\psi }\left(-1/2|3\right). \end{array}$$

## 8. Entangled Two-Qubit Probability Distributions

Now, we consider pure two-qubit states ∣ψ〉 as pure ququart states, with state vectors of the form $|\psi \rangle =|\psi_{1}\rangle |\psi_{2}\rangle$, where $|\psi_{1}\rangle =\left(\begin{array}{c} a\\ b \end{array}\right)$ and $|\psi_{2}\rangle =\left(\begin{array}{c} A\\ B \end{array}\right)$. The density matrix of these states $\rho =|\psi_{1}\rangle |\psi_{2}\rangle \langle \psi_{1}|\langle \psi_{2}|$ is 4 × 4 Hermitian density matrix

(44) $$\rho =\left(\begin{array}{c} a\\ b \end{array}\right)⊗\left(\begin{array}{c} A\\ B \end{array}\right)\left(a^{*},b^{*}\right)⊗\left(A^{*},B^{*}\right).$$

To obtain the probability representations of these states, we introduce the dequantizer operator $\hat{U}\left(X,Y|j,k\right)$, with X=+1/2,−1/2, Y=+1/2,−1/2, j=1,2,3, and k=1,2,3, which are 4 × 4 matrices constructed as a direct product

(45) $$\hat{U}\left(X,Y|j,k\right)=\hat{U}\left(X|j\right)⊗\hat{U}\left(Y|k\right).$$

Since our goal is to calculate entangled probability distributions, we need to obtain traces of density operator product with dequantizers,

$$w_{\psi }\left(X=+1/2,Y=+1/2|3,3\right)=\mathrm{Tr}\left[{\hat{\rho }}_{\;\mathrm{Bell}}\hat{U}\left(+1/2,+1/2|3,3\right)\right],$$

where the density matrix of Bell state reads

(46) $${\hat{\rho }}_{\;\mathrm{Bell}}=\left(\begin{array}{cccc} 1/2 & 0 & 0 & 1/2\\ 0 & 0 & 0 & 0\\ 0 & 0 & 0 & 0\\ 1/2 & 0 & 0 & 1/2 \end{array}\right).$$

Then, we choose the dequantizer $\hat{U}\left(+1/2,+1/2|3,3\right)=\left(\begin{array}{cccc} 1 & 0 & 0 & 0\\ 0 & 0 & 0 & 0\\ 0 & 0 & 0 & 0\\ 0 & 0 & 0 & 0 \end{array}\right)$ and calculate the entangled probability distribution term

(47) $$w_{\mathrm{Bell}}\left(X=+1/2,Y=+1/2|3,3\right)=\mathrm{Tr}\left[\left(\begin{array}{cccc} 1/2 & 0 & 0 & 1/2\\ 0 & 0 & 0 & 0\\ 0 & 0 & 0 & 0\\ 1/2 & 0 & 0 & 1/2 \end{array}\right)\left(\begin{array}{cccc} 1 & 0 & 0 & 0\\ 0 & 0 & 0 & 0\\ 0 & 0 & 0 & 0\\ 0 & 0 & 0 & 0 \end{array}\right)\right]=\frac{1}{2}\;.$$

We list the other dequantizers

(48) $$\hat{U}\left(+1/2,+1/2|3,1\right)=\left(\begin{array}{cccc} \frac{1}{2} & \frac{1}{2} & 0 & 0\\ \frac{1}{2} & \frac{1}{2} & 0 & 0\\ 0 & 0 & 0 & 0\\ 0 & 0 & 0 & 0 \end{array}\right);\;\hat{U}\left(+1/2,+1/2|3,2\right)=\left(\begin{array}{cccc} \frac{1}{2} & -\frac{i}{2} & 0 & 0\\ \frac{i}{2} & \frac{1}{2} & 0 & 0\\ 0 & 0 & 0 & 0\\ 0 & 0 & 0 & 0 \end{array}\right)$$

and

(49) $$\hat{U}\left(+\frac{1}{2},+\frac{1}{2}|1,3\right)=\left(\begin{array}{cccc} \frac{1}{2} & 0 & \frac{1}{2} & 0\\ 0 & 0 & 0 & 0\\ \frac{1}{2} & 0 & \frac{1}{2} & 0\\ 0 & 0 & 0 & 0 \end{array}\right);\;\hat{U}\left(+\frac{1}{2},+\frac{1}{2}|1,2\right)=\left(\begin{array}{cccc} \frac{1}{4} & -\frac{i}{4} & \frac{1}{4} & -\frac{i}{4}\\ \frac{i}{4} & \frac{1}{4} & \frac{1}{4} & \frac{1}{4}\\ \frac{1}{4} & \frac{1}{4} & \frac{1}{4} & -\frac{i}{4}\\ \frac{i}{4} & \frac{1}{4} & \frac{i}{4} & \frac{1}{4} \end{array}\right);$$

they provide the possibility to calculate the other probabilities describing the entangled probability distributions. In a similar way, we can obtain the general result, namely

$$w_{\mathrm{Bell}}\left(X,Y|j,k\right)=\frac{1}{2}\left[{\hat{U}}_{11}\left(X,Y|j,k\right)+{\hat{U}}_{14}\left(X,Y|j,k\right)+{\hat{U}}_{41}\left(X,Y|j,k\right)+{\hat{U}}_{44}\left(X,Y|j,k\right)\right],$$

with $\hat{U}\left(X,Y|j,k\right)$ given by (48) and (49). Explicit values following from this general result are

(50) $$\hat{U}\left(+\frac{1}{2}|2\right)⊗\hat{U}\left(+\frac{1}{2}|2\right)=\left(\begin{array}{cc} \frac{1}{2} & -\frac{i}{2}\\ \frac{i}{2} & \frac{1}{2} \end{array}\right)⊗\left(\begin{array}{cc} \frac{1}{2} & -\frac{i}{2}\\ \frac{i}{2} & \frac{1}{2} \end{array}\right)=\left(\begin{array}{cccc} \frac{1}{4} & -\frac{i}{4} & -\frac{i}{4} & -\frac{1}{4}\\ \frac{i}{4} & \frac{1}{4} & \frac{1}{4} & -\frac{i}{4}\\ \frac{i}{4} & \frac{1}{4} & \frac{1}{4} & -\frac{i}{4}\\ -\frac{1}{4} & \frac{i}{4} & \frac{i}{4} & \frac{1}{4} \end{array}\right),$$

(51) $$\hat{U}\left(+1/2,+1/2|1,1\right)=\left(\begin{array}{cccc} \frac{1}{4} & \frac{1}{4} & \frac{1}{4} & \frac{1}{4}\\ \frac{1}{4} & \frac{1}{4} & \frac{1}{4} & \frac{1}{4}\\ \frac{1}{4} & \frac{1}{4} & \frac{1}{4} & \frac{1}{4}\\ \frac{1}{4} & \frac{1}{4} & \frac{1}{4} & \frac{1}{4} \end{array}\right),$$

(52) $$\hat{U}\left(-1/2|3\right)⊗\hat{U}\left(+1/2|3\right)=\left(\begin{array}{cc} 0 & 0\\ 0 & 1 \end{array}\right)⊗\left(\begin{array}{cc} 1 & 0\\ 0 & 0 \end{array}\right)=\left(\begin{array}{cccc} 0 & 0 & 0 & 0\\ 0 & 0 & 0 & 0\\ 0 & 0 & 1 & 0\\ 0 & 0 & 0 & 0 \end{array}\right),$$

(53) $$\hat{U}\left(-1/2,-1/2|3,3\right)=\left(\begin{array}{cc} 0 & 0\\ 0 & 1 \end{array}\right)⊗\left(\begin{array}{cc} 0 & 0\\ 0 & 1 \end{array}\right)=\left(\begin{array}{cccc} 0 & 0 & 0 & 0\\ 0 & 0 & 0 & 0\\ 0 & 0 & 0 & 0\\ 0 & 0 & 0 & 1 \end{array}\right),$$

(54) $$\hat{U}\left(+1/2,-1/2|3,3\right)=\left(\begin{array}{cc} 1 & 0\\ 0 & 0 \end{array}\right)⊗\left(\begin{array}{cc} 0 & 0\\ 0 & 1 \end{array}\right)=\left(\begin{array}{cccc} 0 & 0 & 0 & 0\\ 0 & 1 & 0 & 0\\ 0 & 0 & 0 & 0\\ 0 & 0 & 0 & 0 \end{array}\right),$$

(55) $$\hat{U}\left(-1/2,+1/2|3,1\right)=\left(\begin{array}{cc} 0 & 0\\ 0 & 1 \end{array}\right)⊗\left(\begin{array}{cc} \frac{1}{2} & \frac{1}{2}\\ \frac{1}{2} & \frac{1}{2} \end{array}\right)=\left(\begin{array}{cccc} 0 & 0 & 0 & 0\\ 0 & 0 & 0 & 0\\ 0 & 0 & \frac{1}{2} & \frac{1}{2}\\ 0 & 0 & \frac{1}{2} & \frac{1}{2} \end{array}\right),$$

(56) $$\hat{U}\left(-1/2,-1/2|3,1\right)=\left(\begin{array}{cc} 0 & 0\\ 0 & 1 \end{array}\right)⊗\left(\begin{array}{cc} \frac{1}{2} & -\frac{1}{2}\\ -\frac{1}{2} & \frac{1}{2} \end{array}\right)=\left(\begin{array}{cccc} 0 & 0 & 0 & 0\\ 0 & 0 & 0 & 0\\ 0 & 0 & \frac{1}{2} & -\frac{1}{2}\\ 0 & 0 & -\frac{1}{2} & \frac{1}{2} \end{array}\right).$$

According to the relationship valid for the conditional probability,

$$w_{\mathrm{Bell}}\left(X,Y|j,k\right)=\mathrm{Tr}\left[{\hat{\rho }}_{\;\mathrm{Bell}}\hat{U}\left(X,Y|j,k\right)\right],$$

we can obtain the following probabilities:

(57) $$\begin{array}{c} w_{\mathrm{Bell}}\left(+1/2,+1/2|3,1\right)=1/4,\;w_{\mathrm{Bell}}\left(+1/2,+1/2|3,2\right)=1/4,\\ w_{\mathrm{Bell}}\left(+1/2,+1/2|1,3\right)=1/4,\;w_{\mathrm{Bell}}\left(+1/2,+1/2|1,2\right)=1/4. \end{array}$$

Analogously, we obtain

(58) $$\begin{array}{ccc} & & w_{\mathrm{Bell}}\left(+1/2,+1/2|2,2\right)=0,\;w_{\mathrm{Bell}}\left(+1/2,+1/2|1,1\right)=1/2,\\ & & w_{\mathrm{Bell}}\left(-1/2,+1/2|3,3\right)=0,\;w_{\mathrm{Bell}}\left(-1/2,-1/2|3,3\right)=1/2,\\ & & w_{\mathrm{Bell}}\left(+1/2,-1/2|3,3\right)=0,\;w_{\mathrm{Bell}}\left(-1/2,+1/2|3,1\right)=1/4. \end{array}$$

Now, we explicitly present all the other dequantizers $\hat{U}\left(X,Y|j,k\right)$ in the form of 4 × 4-matrices; they read

$$\hat{U}\left(+\frac{1}{2},-\frac{1}{2}|1,1\right)=\left(\begin{array}{cccc} \frac{1}{4} & -\frac{1}{4} & \frac{1}{4} & -\frac{1}{4}\\ -\frac{1}{4} & \frac{1}{4} & -\frac{1}{4} & \frac{1}{4}\\ \frac{1}{4} & -\frac{1}{4} & \frac{1}{4} & -\frac{1}{4}\\ -\frac{1}{4} & \frac{1}{4} & -\frac{1}{4} & \frac{1}{4} \end{array}\right),\;\hat{U}\left(+\frac{1}{2},+\frac{1}{2}|1,2\right)=\left(\begin{array}{cccc} \frac{1}{4} & -\frac{i}{4} & \frac{1}{4} & -\frac{i}{4}\\ \frac{i}{4} & \frac{1}{4} & \frac{i}{4} & \frac{1}{4}\\ \frac{1}{4} & -\frac{i}{4} & \frac{1}{4} & -\frac{i}{4}\\ \frac{i}{4} & \frac{1}{4} & \frac{i}{4} & \frac{1}{4} \end{array}\right),$$

$$\hat{U}\left(+\frac{1}{2},-\frac{1}{2}|1,2\right)=\left(\begin{array}{cccc} \frac{1}{4} & \frac{i}{4} & \frac{1}{4} & \frac{i}{4}\\ -\frac{i}{4} & \frac{1}{4} & -\frac{i}{4} & \frac{1}{4}\\ \frac{1}{4} & \frac{i}{4} & \frac{1}{4} & \frac{i}{4}\\ -\frac{i}{4} & \frac{1}{4} & -\frac{i}{4} & \frac{1}{4} \end{array}\right),\;\hat{U}\left(+\frac{1}{2},+\frac{1}{2}|1,3\right)=\left(\begin{array}{cccc} \frac{1}{2} & 0 & \frac{1}{2} & 0\\ 0 & 0 & 0 & 0\\ \frac{1}{2} & 0 & \frac{1}{2} & 0\\ 0 & 0 & 0 & 0 \end{array}\right),$$

$$\hat{U}\left(+\frac{1}{2},-\frac{1}{2}|1,3\right)=\left(\begin{array}{cccc} 0 & 0 & 0 & 0\\ 0 & \frac{1}{2} & 0 & \frac{1}{2}\\ 0 & 0 & 0 & 0\\ 0 & \frac{1}{2} & 0 & \frac{1}{2} \end{array}\right),\;\hat{U}\left(-\frac{1}{2},+\frac{1}{2}|1,1\right)=\left(\begin{array}{cccc} \frac{1}{4} & \frac{1}{4} & -\frac{1}{4} & -\frac{1}{4}\\ \frac{1}{4} & \frac{1}{4} & -\frac{1}{4} & -\frac{1}{4}\\ -\frac{1}{4} & -\frac{1}{4} & \frac{1}{4} & \frac{1}{4}\\ -\frac{1}{4} & -\frac{1}{4} & \frac{1}{4} & \frac{1}{4} \end{array}\right),$$

$$\hat{U}\left(-\frac{1}{2},-\frac{1}{2}|1,1\right)=\left(\begin{array}{cccc} \frac{1}{4} & -\frac{1}{4} & -\frac{1}{4} & \frac{1}{4}\\ -\frac{1}{4} & \frac{1}{4} & \frac{1}{4} & -\frac{1}{4}\\ -\frac{1}{4} & \frac{1}{4} & \frac{1}{4} & -\frac{1}{4}\\ \frac{1}{4} & -\frac{1}{4} & -\frac{1}{4} & \frac{1}{4} \end{array}\right),\;\hat{U}\left(-\frac{1}{2},+\frac{1}{2}|1,2\right)=\left(\begin{array}{cccc} \frac{1}{4} & -\frac{i}{4} & -\frac{1}{4} & \frac{i}{4}\\ \frac{i}{4} & \frac{1}{4} & -\frac{i}{4} & -\frac{1}{4}\\ -\frac{1}{4} & \frac{i}{4} & \frac{1}{4} & -\frac{i}{4}\\ -\frac{i}{4} & -\frac{1}{4} & \frac{i}{4} & \frac{1}{4} \end{array}\right),$$

$$\hat{U}\left(-\frac{1}{2},-\frac{1}{2}|1,2\right)=\left(\begin{array}{cccc} \frac{1}{4} & \frac{i}{4} & -\frac{1}{4} & -\frac{i}{4}\\ -\frac{i}{4} & \frac{1}{4} & \frac{i}{4} & -\frac{1}{4}\\ -\frac{1}{4} & -\frac{i}{4} & \frac{1}{4} & \frac{i}{4}\\ \frac{i}{4} & -\frac{1}{4} & -\frac{i}{4} & \frac{1}{4} \end{array}\right),\;\hat{U}\left(-\frac{1}{2},+\frac{1}{2}|1,3\right)=\left(\begin{array}{cccc} \frac{1}{2} & 0 & -\frac{1}{2} & 0\\ 0 & 0 & 0 & 0\\ -\frac{1}{2} & 0 & \frac{1}{2} & 0\\ 0 & 0 & 0 & 0 \end{array}\right),$$

$$\hat{U}\left(-\frac{1}{2},-\frac{1}{2}|1,3\right)=\left(\begin{array}{cccc} 0 & 0 & 0 & 0\\ 0 & \frac{1}{2} & 0 & -\frac{1}{2}\\ 0 & 0 & 0 & 0\\ 0 & -\frac{1}{2} & 0 & \frac{1}{2} \end{array}\right),\;\hat{U}\left(+\frac{1}{2},+\frac{1}{2}|2,1\right)=\left(\begin{array}{cccc} \frac{1}{4} & \frac{1}{4} & -\frac{i}{4} & -\frac{i}{4}\\ \frac{1}{4} & \frac{1}{4} & -\frac{i}{4} & -\frac{i}{4}\\ \frac{i}{4} & \frac{i}{4} & \frac{1}{4} & \frac{1}{4}\\ \frac{i}{4} & \frac{i}{4} & \frac{1}{4} & \frac{1}{4} \end{array}\right),$$

$$\hat{U}\left(+\frac{1}{2},-\frac{1}{2}|2,1\right)=\left(\begin{array}{cccc} \frac{1}{4} & -\frac{1}{4} & -\frac{i}{4} & \frac{i}{4}\\ -\frac{i}{4} & \frac{1}{4} & \frac{i}{4} & -\frac{i}{4}\\ \frac{i}{4} & -\frac{i}{4} & \frac{1}{4} & -\frac{1}{4}\\ -\frac{i}{4} & \frac{i}{4} & -\frac{1}{4} & \frac{1}{4} \end{array}\right),\;\hat{U}\left(+\frac{1}{2},+\frac{1}{2}|2,2\right)=\left(\begin{array}{cccc} \frac{1}{4} & -\frac{i}{4} & -\frac{i}{4} & -\frac{1}{4}\\ \frac{i}{4} & \frac{1}{4} & \frac{i}{4} & -\frac{i}{4}\\ \frac{i}{4} & \frac{1}{4} & \frac{i}{4} & -\frac{i}{4}\\ -\frac{1}{4} & \frac{i}{4} & \frac{i}{4} & \frac{1}{4} \end{array}\right),$$

$$\hat{U}\left(+\frac{1}{2},-\frac{1}{2}|2,2\right)=\left(\begin{array}{cccc} \frac{1}{4} & \frac{i}{4} & -\frac{i}{4} & \frac{1}{4}\\ -\frac{i}{4} & \frac{1}{4} & -\frac{1}{4} & -\frac{i}{4}\\ \frac{i}{4} & -\frac{1}{4} & \frac{1}{4} & \frac{i}{4}\\ \frac{1}{4} & \frac{i}{4} & -\frac{i}{4} & \frac{1}{4} \end{array}\right)\;\hat{U}\left(+\frac{1}{2},+\frac{1}{2}|2,3\right)=\left(\begin{array}{cccc} \frac{1}{2} & 0 & -\frac{i}{2} & 0\\ 0 & 0 & 0 & 0\\ \frac{i}{2} & 0 & \frac{1}{2} & 0\\ 0 & 0 & 0 & 0 \end{array}\right),$$

$$\hat{U}\left(+\frac{1}{2},-\frac{1}{2}|2,3\right)=\left(\begin{array}{cccc} 0 & 0 & 0 & 0\\ 0 & \frac{1}{2} & 0 & -\frac{i}{2}\\ 0 & 0 & 0 & 0\\ 0 & \frac{i}{2} & 0 & \frac{1}{2} \end{array}\right),\;\hat{U}\left(-\frac{1}{2},+\frac{1}{2}|2,1\right)=\left(\begin{array}{cccc} \frac{1}{4} & \frac{1}{4} & \frac{i}{4} & \frac{i}{4}\\ \frac{1}{4} & \frac{1}{4} & \frac{i}{4} & \frac{i}{4}\\ -\frac{i}{4} & -\frac{i}{4} & \frac{1}{4} & \frac{1}{4}\\ -\frac{i}{4} & -\frac{i}{4} & \frac{1}{4} & \frac{1}{4} \end{array}\right),$$

$$\hat{U}\left(-\frac{1}{2},-\frac{1}{2}|2,1\right)=\left(\begin{array}{cccc} \frac{1}{4} & -\frac{1}{4} & \frac{i}{4} & -\frac{i}{4}\\ -\frac{1}{4} & \frac{1}{4} & -\frac{i}{4} & \frac{i}{4}\\ -\frac{i}{4} & \frac{i}{4} & \frac{1}{4} & -\frac{1}{4}\\ \frac{i}{4} & -\frac{i}{4} & -\frac{1}{4} & \frac{1}{4} \end{array}\right),\;\hat{U}\left(-\frac{1}{2},+\frac{1}{2}|2,2\right)=\left(\begin{array}{cccc} \frac{1}{4} & -\frac{i}{4} & \frac{i}{4} & \frac{1}{4}\\ \frac{i}{4} & \frac{1}{4} & -\frac{1}{4} & \frac{i}{4}\\ -\frac{i}{4} & -\frac{1}{4} & \frac{1}{4} & -\frac{i}{4}\\ \frac{1}{4} & -\frac{i}{4} & \frac{i}{4} & \frac{1}{4} \end{array}\right),$$

$$\hat{U}\left(-\frac{1}{2},-\frac{1}{2}|2,2\right)=\left(\begin{array}{cccc} \frac{1}{4} & \frac{i}{4} & \frac{i}{4} & -\frac{1}{4}\\ -\frac{i}{4} & \frac{1}{4} & \frac{1}{4} & \frac{i}{4}\\ -\frac{i}{4} & \frac{1}{4} & \frac{1}{4} & \frac{i}{4}\\ -\frac{1}{4} & -\frac{i}{4} & -\frac{i}{4} & \frac{1}{4} \end{array}\right),\;\hat{U}\left(-\frac{1}{2},+\frac{1}{2}|2,3\right)=\left(\begin{array}{cccc} \frac{1}{2} & 0 & \frac{i}{2} & 0\\ 0 & 0 & 0 & 0\\ -\frac{i}{2} & 0 & \frac{1}{2} & 0\\ 0 & 0 & 0 & 0 \end{array}\right),$$

$$\hat{U}\left(-\frac{1}{2},-\frac{1}{2}|2,3\right)=\left(\begin{array}{cccc} 0 & 0 & 0 & 0\\ 0 & \frac{1}{2} & 0 & \frac{i}{2}\\ 0 & 0 & 0 & 0\\ 0 & -\frac{i}{2} & 0 & \frac{1}{2} \end{array}\right),\;\hat{U}\left(+\frac{1}{2},-\frac{1}{2}|3,1\right)=\left(\begin{array}{cccc} \frac{1}{2} & -\frac{1}{2} & 0 & 0\\ -\frac{1}{2} & \frac{1}{2} & 0 & 0\\ 0 & 0 & 0 & 0\\ 0 & 0 & 0 & 0 \end{array}\right),$$

$$\hat{U}\left(+\frac{1}{2},+\frac{1}{2}|3,2\right)=\left(\begin{array}{cccc} \frac{1}{2} & -\frac{i}{2} & 0 & 0\\ \frac{i}{2} & \frac{1}{2} & 0 & 0\\ 0 & 0 & 0 & 0\\ 0 & 0 & 0 & 0 \end{array}\right),\;\hat{U}\left(+\frac{1}{2},-\frac{1}{2}|3,2\right)=\left(\begin{array}{cccc} \frac{1}{2} & \frac{i}{2} & 0 & 0\\ -\frac{i}{2} & \frac{1}{2} & 0 & 0\\ 0 & 0 & 0 & 0\\ 0 & 0 & 0 & 0 \end{array}\right),$$

$$\hat{U}\left(+\frac{1}{2},+\frac{1}{2}|3,3\right)=\left(\begin{array}{cccc} 1 & 0 & 0 & 0\\ 0 & 0 & 0 & 0\\ 0 & 0 & 0 & 0\\ 0 & 0 & 0 & 0 \end{array}\right),\;\hat{U}\left(+\frac{1}{2},-\frac{1}{2}|3,3\right)=\left(\begin{array}{cccc} 0 & 0 & 0 & 0\\ 0 & 1 & 0 & 0\\ 0 & 0 & 0 & 0\\ 0 & 0 & 0 & 0 \end{array}\right),$$

$$\hat{U}\left(-\frac{1}{2},+\frac{1}{2}|3,1\right)=\left(\begin{array}{cccc} 0 & 0 & 0 & 0\\ 0 & 0 & 0 & 0\\ 0 & 0 & \frac{1}{2} & \frac{1}{2}\\ 0 & 0 & \frac{1}{2} & \frac{1}{2} \end{array}\right),\;\hat{U}\left(\frac{1}{2},-\frac{1}{2}|3,1\right)=\left(\begin{array}{cccc} 0 & 0 & 0 & 0\\ 0 & 0 & 0 & 0\\ 0 & 0 & \frac{1}{2} & -\frac{1}{2}\\ 0 & 0 & -\frac{1}{2} & \frac{1}{2} \end{array}\right),$$

$$\hat{U}\left(+\frac{1}{2},+\frac{1}{2}|3,2\right)=\left(\begin{array}{cccc} \frac{1}{2} & -\frac{i}{2} & 0 & 0\\ \frac{i}{2} & \frac{1}{2} & 0 & 0\\ 0 & 0 & 0 & 0\\ 0 & 0 & 0 & 0 \end{array}\right),\;\hat{U}\left(+\frac{1}{2},-\frac{1}{2}|3,2\right)=\left(\begin{array}{cccc} \frac{1}{2} & \frac{i}{2} & 0 & 0\\ -\frac{i}{2} & \frac{1}{2} & 0 & 0\\ 0 & 0 & 0 & 0\\ 0 & 0 & 0 & 0 \end{array}\right),$$

$$\hat{U}\left(+\frac{1}{2},+\frac{1}{2}|3,3\right)=\left(\begin{array}{cccc} 1 & 0 & 0 & 0\\ 0 & 0 & 0 & 0\\ 0 & 0 & 0 & 0\\ 0 & 0 & 0 & 0 \end{array}\right),\;\hat{U}\left(+\frac{1}{2},-\frac{1}{2}|3,3\right)=\left(\begin{array}{cccc} 0 & 0 & 0 & 0\\ 0 & 1 & 0 & 0\\ 0 & 0 & 0 & 0\\ 0 & 0 & 0 & 0 \end{array}\right),$$

$$\hat{U}\left(-\frac{1}{2},+\frac{1}{2}|3,2\right)=\left(\begin{array}{cccc} 0 & 0 & 0 & 0\\ 0 & 0 & 0 & 0\\ 0 & 0 & \frac{1}{2} & -\frac{i}{2}\\ 0 & 0 & \frac{i}{2} & \frac{1}{2} \end{array}\right),\;\hat{U}\left(-\frac{1}{2},-\frac{1}{2}|3,2\right)=\left(\begin{array}{cccc} 0 & 0 & 0 & 0\\ 0 & 0 & 0 & 0\\ 0 & 0 & \frac{1}{2} & \frac{i}{2}\\ 0 & 0 & -\frac{i}{2} & \frac{1}{2} \end{array}\right).$$

Using the dequantizers obtained, in view of relationship (50), we calculate all conditional probabilities describing the entangled Bell state. We calculate and present all probabilities belonging to entangled probability distributions describing the Bell state (50). In addition to (57) and (58) already obtained, we present the rest, namely

(59) $$\begin{array}{ccc} & & w_{\mathrm{Bell}}\left(+1/2,-1/2|1,1\right)=0;\;\;w_{\mathrm{Bell}}\left(+1/2,-1/2|1,2\right)=1/4;\;\;w_{\mathrm{Bell}}\left(+1/2,-1/2|1,3\right)=1/4;\\ & & w_{\mathrm{Bell}}\left(-1/2,-1/2|1,1\right)=1/2;\;\;w_{\mathrm{Bell}}\left(-1/2,+1/2|1,2\right)=1/4;\;\;w_{\mathrm{Bell}}\left(-1/2,-1/2|1,2\right)=1/4;\\ & & w_{\mathrm{Bell}}\left(-1/2,+1/2|1,3\right)=1/4;\;\;w_{\mathrm{Bell}}\left(-1/2,-1/2|1,3\right)=1/4;\;\;w_{\mathrm{Bell}}\left(+1/2,+1/2|2,1\right)=1/4;\\ & & w_{\mathrm{Bell}}\left(+1/2,-1/2|2,1\right)=1/4;\;\;w_{\mathrm{Bell}}\left(+1/2,-1/2|2,2\right)=1/2;\;\;w_{\mathrm{Bell}}\left(+1/2,+1/2|2,3\right)=1/4;\\ & & w_{\mathrm{Bell}}\left(+1/2,-1/2|2,3\right)=1/4;\;\;w_{\mathrm{Bell}}\left(-1/2,+1/2|2,1\right)=1/4;\;\;w_{\mathrm{Bell}}\left(-1/2,-1/2|2,1\right)=1/4;\\ & & w_{\mathrm{Bell}}\left(-1/2,+1/2|2,2\right)=1/2;\;\;w_{\mathrm{Bell}}\left(-1/2,-1/2|2,2\right)=0;\;\;w_{\mathrm{Bell}}\left(-1/2,+1/2|2,3\right)=1/4;\\ & & w_{\mathrm{Bell}}\left(-1/2,-1/2|2,3\right)=1/4;\;\;w_{\mathrm{Bell}}\left(-1/2,+1/2|1,1\right)=0;\;\;w_{\mathrm{Bell}}\left(+1/2,-1/2|3,1\right)=1/4;\\ & & w_{\mathrm{Bell}}\left(-1/2,-1/2|3,1\right)=1/4;\;\;w_{\mathrm{Bell}}\left(+1/2,-1/2|3,2\right)=1/4;\;\;w_{\mathrm{Bell}}\left(+1/2,+1/2|3,3\right)=1/2;\\ & & w_{\mathrm{Bell}}\left(+1/2,-1/2|3,3\right)=0;\;\;w_{\mathrm{Bell}}\left(-1/2,-1/2|3,2\right)=1/4. \end{array}$$

For any state, the obtained probabilities must satisfy the equality

(60) $$w\left(+1/2,+1/2|j,k\right)+w\left(-1/2,+1/2|j,k\right)=w\left(+1/2|k\right);$$

it can be checked for Bell state, and this means that the sums of conditional probabilities in the left-hand side of this equality should be equal to each other for all three values of j=1,2,3 and for all three values of k=1,2,3. Also, it is worth mentioning that it is a nice way to check correctness of the calculations, which we just used.

Thus, we checked the new obtained equalities for entangled states, and, in the next section, we consider separable states of two qubits.

## 9. Separable Probability Distributions

We introduce a new notion of separable probability distributions on the example of the probability distribution describing two-qubit states with the density matrix of the form $\rho_{S}=\left(\begin{array}{cccc} 1/2 & 0 & 0 & 0\\ 0 & 0 & 0 & 0\\ 0 & 0 & 0 & 0\\ 0 & 0 & 0 & 1/2 \end{array}\right)$; it corresponds to the convex sum of two matrices $\rho_{1}⊗\rho_{1}$, where $\rho_{1}=\left(\begin{array}{cc} 1 & 0\\ 0 & 0 \end{array}\right)$, and $\rho_{2}⊗\rho_{2}$, where $\rho_{2}=\left(\begin{array}{cc} 0 & 0\\ 0 & 1 \end{array}\right)$, namely $\rho_{S}=\frac{1}{2}\left[\rho_{1}⊗\rho_{1}+\rho_{2}⊗\rho_{2}\right]$, and it is the density matrix of the separable state under discussion. Then, for the separable state with the density matrix $\rho_{S}$, we have the probabilities

(61) $$\begin{array}{ccc} & & w_{S}\left(-1/2,-1/2|3,3\right)=1/2;\;\;w_{S}\left(+1/2,+1/2|3,1\right)=1/4;\;\;w_{S}\left(+1/2,+1/2|3,2\right)=1/4;\\ & & w_{S}\left(+1/2,+1/2|1,3\right)=1/4;\;\;w_{S}\left(+1/2,+1/2|1,2\right)=1/4;\;\;w_{S}\left(+1/2,+1/2|2,2\right)=1/4;\\ & & w_{S}\left(+1/2,+1/2|1,1\right)=1/4;\;\;w_{S}\left(-1/2,+1/2|3,3\right)=0;\;\;w_{S}\left(-1/2,-1/2|3,1\right)=1/4;\\ & & w_{S}\left(+1/2,-1/2|1,1\right)=1/4;\;\;w_{S}\left(+1/2,-1/2|1,2\right)=1/4;\;\;w_{S}\left(+1/2,-1/2|1,3\right)=1/4;\\ & & w_{S}\left(-1/2,+1/2|1,1\right)=1/4;\;\;w_{S}\left(-1/2,-1/2|1,1\right)=1/4;\;\;w_{S}\left(-1/2,-1/2|1,2\right)=1/4;\\ & & w_{S}\left(-1/2,-1/2|1,3\right)=1/4;\;\;w_{S}\left(+1/2,+1/2|2,1\right)=1/4;\;\;w_{S}\left(+1/2,-1/2|2,1\right)=1/4;\\ & & w_{S}\left(+1/2,+1/2|2,2\right)=1/4;\;\;w_{S}\left(+1/2,-1/2|2,2\right)=1/4;\;\;w_{S}\left(+1/2,+1/2|2,3\right)=1/4;\\ & & w_{S}\left(+1/2,-1/2|2,3\right)=1/4;\;\;w_{S}\left(-1/2,+1/2|2,1\right)=1/4;\;\;w_{S}\left(-1/2,-1/2|2,1\right)=1/4;\\ & & w_{S}\left(-1/2,+1/2|2,2\right)=1/4;\;\;w_{S}\left(-1/2,-1/2|2,2\right)=1/4;\;\;w_{S}\left(-1/2,+1/2|2,3\right)=1/4;\\ & & w_{S}\left(-1/2,-1/2|2,3\right)=1/4;\;\;w_{S}\left(+1/2,-1/2|3,1\right)=1/4;\;\;w_{S}\left(+1/2,+1/2|3,2\right)=1/4;\\ & & w_{S}\left(+1/2,-1/2|3,2\right)=1/4;\;\;w_{S}\left(+1/2,+1/2|3,3\right)=1/2;\;\;w_{S}\left(+1/2,-1/2|3,3\right)=0;\\ & & w_{S}\left(+1/2,-1/2|3,1\right)=1/4;\;\;w_{S}\left(-1/2,+1/2|3,2\right)=1/4;\;\;w_{S}\left(-1/2,-1/2|3,2\right)=1/4. \end{array}$$

Let us compare two probability distributions under consideration, i.e., look for the difference between $w_{\mathrm{Bell}}\left(X,Y|j,k\right)$ and $w_{S}\left(X,Y|j,k\right)$; the first one is the entangled probability distribution obtained using the normalized superposition of two qubit states $|\psi_{1}\rangle =\left(\begin{array}{c} 1\\ 0 \end{array}\right)⊗\left(\begin{array}{c} 1\\ 0 \end{array}\right)$ and $|\psi_{2}\rangle =\left(\begin{array}{c} 0\\ 1 \end{array}\right)⊗\left(\begin{array}{c} 0\\ 1 \end{array}\right)$, with the Bell density matrix ${\hat{\rho }}_{\;\mathrm{Bell}}$ given by (46), and the second one is the separable probability distribution describing the normalized separable state $\frac{1}{2}\left[\left(|\psi_{1}\rangle \langle \psi_{1}|\right)+\left(|\psi_{2}\rangle \langle \psi_{2}|\right)\right]$, with the density matrix $\rho_{S}=\frac{1}{2}\left(\begin{array}{cccc} 1 & 0 & 0 & 0\\ 0 & 0 & 0 & 0\\ 0 & 0 & 0 & 0\\ 0 & 0 & 0 & 1 \end{array}\right)$. The difference in the entangled probability distribution and separable probability distribution contains the influence of the superposition term contribution; it reads

(62) $$\begin{array}{c} w_{\mathrm{Bell}}\left(X,Y|j,k\right)-w_{S}\left(X,Y|j,k\right)=\frac{1}{2}\left[\hat{U}{\left(X,Y|j,k\right)}_{14}+\hat{U}{\left(X,Y|j,k\right)}_{41}\right], \end{array}$$

where $\hat{U}\left(X,Y|j,k\right)$ are dequantizer operators. For example,

(63) $$\begin{array}{c} w_{\mathrm{Bell}}\left(+1/2,+1/2|3,3\right)-w_{S}\left(+1/2,+1/2|3,3\right)=0, \end{array}$$

(64) $$\begin{array}{c} w_{\mathrm{Bell}}\left(-1/2,-1/2|1,1\right)-w_{S}\left(-1/2,-1/2|1,1\right)=1/4. \end{array}$$

## 10. Conclusions

Concluding, we summarize the results presented in this paper.

During a hundred years of development of quantum mechanics, quite a new notion of the state of a mechanical object, like a particle, using a complex wave function or vector in the Hilbert space and the density operator acting in the Hilbert space, was introduced. Nevertheless, the dream of the researchers was to find a classically clear object, like the notion of probability distribution available in classical statistical mechanics, to describe quantum states. For many decades, this dream created a similar but different notion to the Wigner function or the other quasiprobability distributions to describe quantum states. Only recently, the standard probability distribution method for describing quantum states was suggested, and, in this work, we studied the application of this method to the state of qubits.

We explicitly obtained the probability distributions of qubit states for specific interesting states, such as entangled Bell states. We constructed the probability distribution representation of the two-spin-1/2 state and found the conditional probability distribution describing all 36 probabilities of both spin-1/2 projections onto different directions in the space. It is worth mentioning that the Bell state of two spin-1/2 contains correlations of two spin-subsystems associated with the superposition structure of the wave function describing the spins. Also, we constructed the probability representation describing the separable state of spins without the superposition structure and without such correlation.

We clarified the difference in the obtained probability distributions, which we called entangled probability distributions and separable probability distributions; such a notion has never been considered in standard probability theory because neither in classical physics nor in the other science areas were these probability distributions known and discussed. The details of these probability distributions, like different entropies and entropic inequalities, as well as explicit relations for different conditional probability distributions describing qubit states, will be considered in future publications. Since we constructed different quantizer–dequantizer operators for systems of two qubits, we can consider the relations of different probability representations of the same ququart states using probability distributions corresponding to the different quantizer–dequantizer operators [37], which are associated with the traces of dequantizer products. This means that there exist different probability distribution representations of one density operator of the ququart state related to the other by means of the probabilities determined by the trace of product of different ququart dequantizers; these relations will be studied in future publications.

It is worth mentioning recent books [38,39,40] in which contributions of the international community into establishing and developing the approach described in our paper are presented. Also, we should point out that new directions in theoretical research based on introduced probability representation of quantum states were used in quantum cosmology [41,42] and were recently applied in mathematical methods of group theory [43,44].

## Data Availability

The data presented in this study are available on request from the corresponding author.
